# Is there an association between pelvic pain and gender-affirming testosterone therapy in trans masculine adolescents? An exploratory cross-sectional study

**DOI:** 10.1080/26895269.2024.2392685

**Published:** 2024-08-20

**Authors:** Dehlia Moussaoui, Monsurul Hoq, Charlotte V. Elder, Sonia R. Grover, Michele A. O'Connell, Ken C. Pang

**Affiliations:** a Department of Paediatric and Adolescent Gynaecology, The Royal Children’s Hospital Melbourne, Parkville, Victoria, Australia; b Murdoch Children’s Research Institute, Parkville, Victoria, Australia; c Department of Paediatrics, University of Melbourne, Melbourne, Victoria, Australia; d Department of Endocrinology and Diabetes, The Royal Children’s Hospital Melbourne, Parkville, Victoria, Australia; e Department of Adolescent Medicine, The Royal Children’s Hospital Melbourne, Parkville, Victoria, Australia

**Keywords:** Adolescent, pelvic pain, sexual and gender minorities, testosterone, transgender

## Abstract

**Background:**

High rates of pelvic pain have been reported in trans individuals on testosterone, but the pathophysiology of pain and the potential role of testosterone are unclear.

**Aims:**

The aim of this exploratory study was to determine whether the proportion of pelvic pain in trans adolescents is different among those on testosterone therapy compared to those who are not. Secondary objectives were to determine the characteristics, potential contributing factors, impact, and treatment of pelvic pain in trans adolescents.

**Methods:**

An online cross-sectional study about pelvic pain was conducted among trans individuals assigned female at birth, aged 12 and over, and who had sought care at our institution since 2017.

**Results:**

Among 102 participants, 79 (77.5%) reported having experienced pelvic pain over the last 6 months. The proportion of individuals who reported pelvic pain was lower in individuals on testosterone (*n* = 43/62, 69.4%) compared to those not on testosterone (*n* = 36/40, 90%), with a difference in proportion of −20.6% (95% CI −35.4 to −5.9%, *p* = 0.006). Half of participants reported school or work absenteeism and two thirds missed extracurricular activities because of pelvic pain. A wide range of treatment options were used, with variable rates of reported effectiveness.

**Conclusion:**

Pelvic pain is frequently reported by trans adolescents. This exploratory study found a lower proportion of pelvic pain in trans adolescents using testosterone therapy compared to those who were not. Nevertheless, this study was limited by a small number of participants, its cross-sectional nature, and the risk of recruitment bias, thus limiting its generalizability. Longitudinal studies are required to better understand the development and evolution of pelvic pain in trans adolescents.

## Introduction

Trans and gender diverse (hereafter, trans) individuals who were assigned female at birth may experience gynecological conditions similar to those of cisgender females, including dysmenorrhea and pelvic pain (Moussaoui et al., 2023). Pelvic pain refers to pain located to the pelvis and may be caused by gynecological, musculoskeletal, neurological, bowel and urinary tract dysfunction (“Chronic Pelvic Pain,” 2020; Lamvu et al., 2021). Chronic pelvic pain may be continuous or recurrent, and includes cyclical conditions such as dysmenorrhea, which is pain associated with menstruation (“Chronic Pelvic Pain,” 2020; *International Association for the Study of Pain. Classification of Chronic Pain, Second Edition (Revised). Published 2020.*). Dysmenorrhea is estimated to affect 71*–*92% of the cisgender female population (Armour et al., 2019, 2020; Schoep et al., 2019). Prevalence of pelvic pain is mostly reported after exclusion of cyclical causes, and in this context ranges between 5.7–26.6% in cisgender female adults (Ahangari, 2014), and 14–55% in cisgender female adolescents and young adults (Armour et al., 2020; Sasamoto et al., 2023; Tabaac et al., 2022).

Recently, high rates of pelvic pain have been reported in adult trans cohorts, and the use of testosterone gender-affirming hormone therapy in this population has been suggested as a potential contributing factor to pelvic pain, potentially through changes to the pelvic floor musculature and/or endometrial activity (Grimstad et al., 2020, 2023; Zwickl et al., 2023). In a retrospective case series, Grimstad et al. observed that 57.7% of trans men on testosterone who were requesting gender-affirming hysterectomy reported pelvic pain, but the authors acknowledged likely recruitment biases given hysterectomies can be used to treat pelvic pain (Grimstad et al., 2019). The same group subsequently reported that 69.4% of trans men experienced new-onset pelvic pain after starting testosterone but the authors emphasized the preliminary nature of their study, which relied upon an exploratory cross-sectional survey of participants recruited *via* convenience sampling (Grimstad et al., 2020). Similarly, Zwickl et al. reported that 72.2% of trans men on testosterone experience pelvic pain (Zwickl et al., 2023) but, like the earlier Grimstad et al papers, this study was not designed to provide a true prevalence rate given likely recruitment biases. In addition, none of these studies included a comparison group of trans individuals who were not on testosterone, thus limiting their ability to determine whether or not testosterone is actually associated with an increased risk of pelvic pain. In keeping with these studies potentially providing an over-estimate of pelvic pain, a more recent study by Grimstad et al. observed that only 36% of patients receiving testosterone reported pelvic pain (compared to a rate of 15% prior to starting testosterone) (Grimstad et al., 2023). However, these results were based on a retrospective chart review, which may have under-estimated the true prevalence rate both before and after testosterone initiation.

There is currently limited information available on pelvic pain in trans adolescents. This is an important topic to explore because pelvic pain is the leading cause of school absenteeism in adolescents assigned female at birth more generally, and has negative consequences on their quality of life (Gallagher et al., 2018; Munro et al., 2023). In one study, a retrospective chart review was undertaken specifically to study the presentation of dysmenorrhea and endometriosis in transmasculine adolescents (Shim et al., 2020). In a separate retrospective chart review, it was observed that 23.4% of trans adolescents on testosterone had reported pelvic pain to their clinicians (Moussaoui et al., 2024). The authors also described characteristics and treatment of pelvic pain within this group, and observed that use of menstrual suppression was associated with an increased risk of pelvic pain. However, the retrospective nature of this study was likely to have failed to fully capture the prevalence and nature of pelvic pain in this population.

Given the limited data on prevalence and characteristics of pelvic pain in trans adolescents, we designed this exploratory study aiming to determine whether the proportion of individuals experiencing pelvic pain in trans adolescents is different among those using testosterone therapy compared to those who are not. Secondary objectives were to determine the characteristics, potential contributing factors, impact and treatment of pelvic pain in trans adolescents.

## Methods

### Study design

An online cross-sectional survey was conducted between December 2022 and August 2023.

### Study setting

The study was conducted at The Royal Children’s Hospital Gender Service (RCHGS), a pediatric tertiary referral clinic for trans children and adolescents, based in Melbourne, Australia.

### Participants

The study participants were eligible to participate if they were: trans and gender diverse individuals assigned female at birth, aged 12 and over, who had sought care at our institution since 2017.

### Recruitment

An email was sent to eligible individuals within the Gender Service clinical database (*n* = 925) in December 2022. Emails were sent directly to the young person if their email address was available and they were aged more than 18 at the time of the survey or they had presented as a “mature minor” (i.e. without the knowledge or consent of their guardians) (*N* = 123). Emails were sent to the parents/guardians for other cases (*N* = 802). The email contained a link to an information statement, consent form, and the survey. By sharing the link with their child, the parents provided implied consent. The survey was also advertised in the waiting room of the Gender Service *via* a flyer with a QR code. In addition, the survey was advertised in the Gender Service Newsletter which was sent by email in June 2023 to the parents/guardians of all current patients of the service, to patients presenting as “mature minors,” and to those patients who had been discharged and consented to be contacted about future studies associated with the Gender Service. The survey was titled “Survey on pelvic pain in trans adolescents” and information about the study provided in the recruitment material stated that the survey was about pelvic pain, while still encouraging those without pelvic pain to participate. Recruitment stopped in August 2023.

### Survey design

A 41-question online survey instrument was used to collect data from study participants. The questionnaire was developed by the authors (see Supplementary Material), since there is no validated questionnaire about pelvic pain in trans individuals. Questions included multiple choice, Likert-scale questions as well as open-ended questions. Participants could opt-out of providing an answer to questions, except for the one asking about whether they experience pelvic pain. The online survey was designed in REDCap. Healthcare providers and trans individuals, who were not otherwise involved in the research project, piloted the survey and provided comments and suggestions for revisions before the survey was finalized. The survey was designed to be completed in less than 10 min. There was no financial compensation offered to participants.

### Survey measures

Participants were asked about past or current treatment with testosterone, including formulation and length of use. Participants were asked whether they had experienced pelvic pain over the last 6 months or not. Pelvic pain was defined as abdominal pain in the lower half of the abdomen (i.e. below the navel) with a drawing used to facilitate this definition. If the participant reported pelvic pain, further questions were asked regarding the description of this pain, its intensity (using a Likert scale where 0 is no pain and 10 is the worst possible pain), location (using a picture of the abdomen wall) and impact on participation at school, work or extracurricular activities. Possible treatments for pelvic pain were listed and participants were asked to identify those that they had tried and whether these had helped or not. Masturbation, penetrative sexual activity and orgasm were explored as potential triggers for pelvic pain. In those who reported being on testosterone and having pelvic pain, timing and evolution of pelvic pain while on testosterone were explored. Participants were asked about periods and whether they ever had experienced period pain. Questions were asked about use of puberty blockers and menstrual suppression. In those on testosterone, questions were asked about breakthrough bleeding. Information was also collected about gender identity, sexual orientation, age and current medical conditions (including mental health conditions and chronic pain conditions).

### Statistical analyses

Statistical analyses were performed using Stata 17.0. Categorical data were reported as number and percentage, and continuous data as mean (standard deviation) or median (interquartile range (IQR)) depending on their distribution. Differences in proportion of individuals with pelvic pain between those who were using testosterone and those who were not, were calculating using binomial regression. Results in the regression model were adjusted for medication use for menstrual suppression (excluding testosterone), given the previous observation that menstrual suppression is associated with a higher risk of pelvic pain (Moussaoui et al., 2024). Differences in proportion of individuals with pelvic pain between those who were using menstrual suppression and those who were not, were calculated using binomial regression, and results were adjusted for testosterone use. A test of two proportions was performed to compare pain characteristics between individuals who were using testosterone and those who were not.

### Ethics

This study was approved by the local Hospital Human Research Ethics Committee (#88435).

## Results

### Participant characteristics

102 young people completed the survey, and their clinical characteristics are provided in Table 1.

**Table 1.** Participant characteristics.

	Participants not on testosterone, *n* = 40	Participants on testosterone, *n* = 62	Total participants, *n* = 102
Age (years), mean (SD)	16.4 (1.9)	19.2 (1.9)	18.1 (2.3)
Gender identity, *n* (%)			
Male	26/40 (65)	57/62 (91.9)	83/102 (81.4)
Non-binary	14/40 (35)	5/62 (8.1)	19/102 (18.6)
Current use of testosterone, *n* (%)			62/102 (60.8)
Topical formulation	–	10/62 (16.1)	–
Short-acting injection (*testosterone enanthate*)	–	0 (0)	–
Long-acting injection (*testosterone undecanoate*)	–	52/62 (83.9)	–
Duration on testosterone, *n* (%)			
Less than 12 months	–	13/62 (21)	–
Between 12 and 24 months	–	18/62 (29)	–
More than 24 months	–	31/62 (50)	–
Ever used GnRHa, *n* (%)^a^	2/39 (5.1)	3/60 (5)	5/99 (5)
Current use of menstrual suppression, *n* (%)^a^^,^^b^	28/39 (71.8)	22/58 (37.9)	50/97 (51.5)
Oral progestin	15/39 (38.5)	11/58 (19)	26/97 (26.8)
IM medroxyprogesterone acetate	1/39 (2.6)	2/58 (3.4)	3/97 (3.1)
Levonorgestrel IUD	3/39 (7.7)	4/58 (6.9)	7/97 (7.2)
Etonogestrel	2/39 (5.1)	4/58 (6.9)	6/97 (6.2)
Oral combined contraceptive pill	8/39 (20.5)	2/58 (3.4)	10/97 (10.3)
Ever experienced dysmenorrhea, *n* (%)^a^	37/39 (94.9)	58/59 (98.3)	95/98 (96.9)
Comorbidities - mental health, *n* (%)			
PTSD	7/40 (17.5)	9/62 (14.5)	16/102 (15.7)
ASD	12/40 (30)	18/62 (29)	30/102 (29.4)
Depression	24/40 (60)	43/62 (69.4)	67/102 (65.7)
Anxiety	28/40 (70)	46/62 (74.2)	74/102 (72.6)
Comorbidities - chronic pain conditions, *n* (%)			
Endometriosis	0	1/62 (1.6)	1/102 (1)
Fibromyalgia	0	3/62 (4.8)	3/102 (2.9)
Migraine	4/40 (10)	5/62 (8.1)	9/102 (8.8)
Back pain	9/40 (22.5)	7/62 (11.3)	16/102 (15.7)
BMI (kg/m2), median (IQR)	26.1 (21.1 to 33)	24.2 (22.1 to 31.7)	24.5 (21.8 to 31.9)

SD = standard deviation, GnRHa = gonadotropin-releasing hormone agonist, IM = intramuscular, IUD = intra-uterine device, ASD = autism spectrum disorder, PTSD = post-traumatic stress disorder, BMI = body mass index, IQR = interquartile range

^a^Total is less than 102 due to missing data.

^b^Two individuals were using two concomitant hormonal medications for menstrual suppression (levonorgestrel IUD and oral progestin, and levonorgestrel IUD and IM medroxyprogesterone acetate).

Testosterone was used by 60.8% (*n* = 62/102) of the participants, with long-acting injectable preparations (testosterone undecanoate) being the most common formulation used by participants (83.9%, *n* = 52/62) and the majority (79%, *n* = 49/62) of users having been on treatment for at least 1 year. One participant had used testosterone in the past but stopped it more than 6 months ago, therefore they were included in the group “no testosterone” for analysis.

A history of dysmenorrhea was reported by 96.9% (*n* = 95/98) of the total cohort, with a median score of 7 (IQR 5–8) on a pain scale 0–10. Mental health conditions were commonly reported by participants, with almost three quarters reporting suffering from anxiety, two thirds from depression and a third from autism spectrum disorder (Table 1).

### Proportion of pelvic pain

Among the study participants, 77.5% (*n* = 79/102) reported having pelvic pain in the six months prior to survey completion.

### Association between pelvic pain and testosterone

The proportion of those who had experienced pelvic pain in the past six months among those who were using testosterone was 69.4% (*n* = 43/62) and among those who were not using testosterone was 90% (*n* = 36/40), giving a difference in proportion of −20.6% (95% CI −35.4 to −5.9%, *p* = 0.006). After taking into account medication use for menstrual suppression, the adjusted difference in proportion of pelvic pain was −18.8% (95% CI −34.1 to −3.5%, *p* = 0.016) for individuals using testosterone compared to those who were not.

Among those who were on testosterone and who reported pelvic pain, most (83.3%, *n* = 35/42) said that pain was already present before they started testosterone. In 38.1% (*n* = 16/42), pain had not changed since the initiation of testosterone, in 21.4% (*n* = 9/42) pain was reported as improving, and in 23.8% (*n* = 10/42) pain was reported as worsening after testosterone initiation. Only 16.7% (*n* = 7/42) said that pain first occurred after they started testosterone. Among those using testosterone, 16.1% (*n* = 10/62) reported breakthrough bleeding on testosterone, and most of those experienced pelvic pain (80%, *n* = 8/10).

### Association between pelvic pain and medication use for menstrual suppression

The proportion of those who had experienced pelvic pain in the past six months was larger among those who were using menstrual suppression compared to those who were not using menstrual suppression (90%, *n* = 45/50 vs 65.4%, *n* = 34/52, difference in proportion of 24.6% percentage points, 95% CI 9.2% to 40%, *p* = 0.002). After taking into account testosterone status, however, the adjusted difference in proportion of pelvic pain was only 2.6% (95% CI −20.7 to 26%, *p* = 0.826) higher in individuals using menstrual suppression compared to those who were not.

### Pelvic pain characteristics

**Figure 1.**
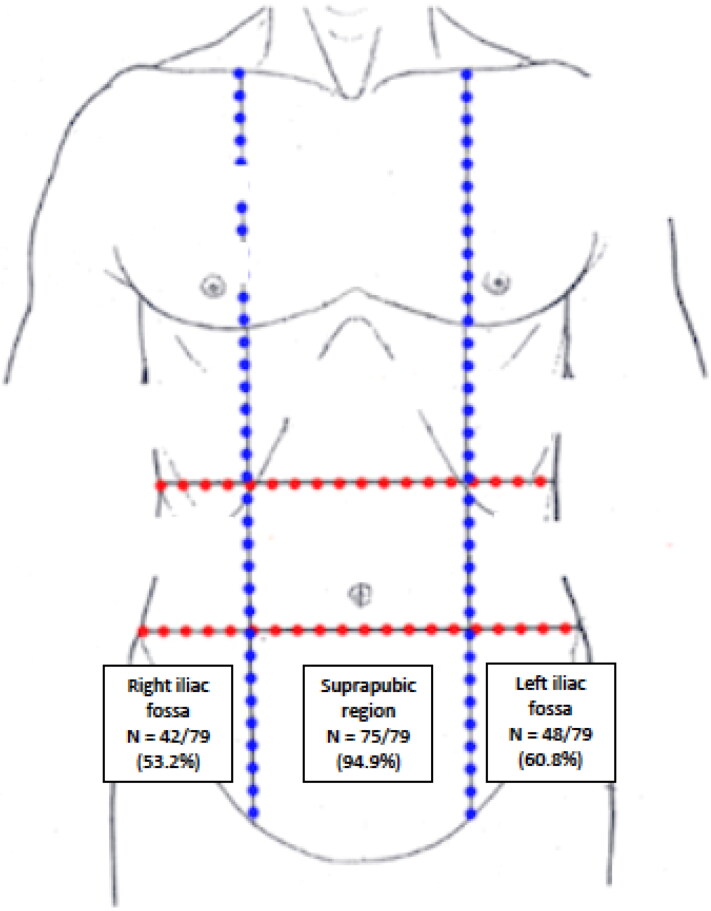
Location of pelvic pain. Prevalence of pelvic pain according to locations reported by participants. Image adapted from https://teachmeanatomy.info/abdomen/muscles/abdominal-wall/

Participants most commonly reported pain in the suprapubic region (94.9%, *n* = 75/79), left iliac fossa (60.8%, *n* = 48/79) and right iliac fossa (53.2, *n* = 42/79) (Figure 1). Pelvic pain characteristics among survey participants are provided in Table 2.

**Table 2.** Pelvic pain characteristics.

	Participants not on testosterone, *n* = 36	Participants on testosterone, *n* = 43	Total participants with pelvic pain, *n* = 79	Difference in proportion (95% CI)	*P*-value
Pain description, *n* (%)					
Cramping	32/36 (88.9)	38/43 (88.4)	70/79 (88.6)	−0.5 (−14.6 to 13.5)	0.943
Aching	21/36 (58.3)	24/43 (55.8)	45/79 (57)	−2.5 (−24.4 to 19.4)	0.822
Sharp	17/36 (47.2)	17/43 (39.5)	34/79 (43)	−7.7 (−29.6 to 14.2)	0.492
Stabbing	14/36 (38.9)	14/43 (32.6)	28/79 (35.4)	−6.3 (−27.5 to 14.9)	0.558
Throbbing	9/36 (25)	18/43 (41.9)	27/79 (34.2)	16.9 (−3.6 to 37.3)	0.116
Burning	5/36 (13.9)	4/43 (9.3)	9/79 (11.4)	−4.6 (−18.8 to 9.7)	0.523
Pain severity (0–10 scale), *n* (%)^a^					
0–2	1/35 (2.9)	5/43 (11.6)	6/78 (7.7)	8.9 (−2.1 to 19.8)	0.139
3–4	4/35 (11.4)	8/43 (18.6)	12/78 (15.4)	7.5 (−8.0 to 23.0)	0.355
5–6	11/35 (31.4)	12/43 (27.9)	23/78 (29.5)	−2.6 (−22.8 to 17.5)	0.796
7–8	18/35 (51.4)	17/43 (39.5)	35/78 (44.9)	−10.5 (−32.4 to 11.5)	0.351
9–10	1/35 (2.9)	1/43 (2.3)	2/78 (2.6)	−0.5 (−7.5 to 6.6)	0.899
Pain with sexual activity, *n* (%)^a^					
Masturbation	8/21 (38.1)	18/37 (48.6)	26/58 (44.8)	10.6 (−15.7 to 36.8)	0.437
Vaginal or anal penetration	13/17 (76.5)	18/24 (75)	31/41 (75.6)	−1.5 (−28.1 to 25.1)	0.914
Orgasm	4/17 (23.5)	20/34 (58.8)	24/51 (47.1)	35.3 (9.2 to 61.4)	0.017

^a^Total number is less than 79 because of missing data or option to answer “not applicable.”

On a scale from 0–10, the median pelvic pain score was 6 (IQR 5–7). Lower pain scores (0–2/10 and 3–4/10) were more common in individuals using testosterone compared to those who were not, while higher pain scores (5–6/10 and 7–8/10) were more common in individuals not using testosterone compared to those using it (Table 2), although these differences were not statistically significant. More than half of participants reported school or work absenteeism because of pain (57%, *n* = 45/79). In addition, pain prevented 69.6% (*n* = 55/79) of the individuals from participating in extracurricular activities.

Pain was most commonly described as cramping (88.6%, *n* = 70/79), followed by aching (57%, *n* = 45/79) and/or sharp (43%, *n* = 34/79). The description of pain was similar between those who used testosterone and those who did not (Table 2). Sexual activity was a common trigger of pain, with 44.8% (*n* = 26/58) of the participants reporting pain with masturbation, 47.1% (*n* = 24/51) with orgasm, and 75.6% (*n* = 31/41) with vaginal or anal penetration. The percentage of participants that reported pain with penetration was similar between those who used testosterone (75%, *n* = 18/24) and those who did not (76.5%, *n* = 13/17). The proportion of participants who reported pain with orgasm was higher in individuals using testosterone compared with those who were not using testosterone (58.8%, *n* = 20/34 vs 23.5%, *n* = 4/17; difference in proportion: 35.3%, 95% CI 9.2 to 61.4%, *p* = 0.017).

### Pelvic pain treatment

The most common pharmacological strategies to relieve pelvic pain (Table 3) were paracetamol (81%, *n* = 64/79), non-steroidal anti-inflammatory drugs (NSAIDs) (60.8%, *n* = 48/79), and oral progestins (26.6%, *n* = 21/79). Around 50–60% of participants who tried these agents reported them as effective. Non-pharmacological strategies, including heat (64.6%, *n* = 51/79) and physical exercise (49.4%, *n* = 39/79), were also commonly tried, with heat reported to be effective by almost 69% (*n* = 35/51) of respondents who used it, which was much higher than for those who tried exercise (13%, *n* = 5/39). A variety of other strategies were also described less commonly, including IM medroxyprogesterone acetate (6.3%, *n* = 5/79), levonorgestrel IUD (8.9%, *n* = 7/79), morphine (5.1%, *n* = 4/79), pelvic floor physiotherapy (5.1%, *n* = 4/79) and buscopan (2.5%, *n* = 2/79). Of these other strategies, pelvic floor physiotherapy (50%, *n* = 2/4) and buscopan (100%, *n* = 2/2) had the highest levels of reported effectiveness but only a small number of participants tried these options.

**Table 3.** Treatment strategies for pelvic pain.

	Participants with pelvic pain who tried the medication, *n* (%)	Participants who tried the medication and found it effective, *n* (%)
Paracetamol	64/79 (81)	35/64 (54.7)
Heat	51/79 (64.6)	35/51 (68.6)
NSAID	48/79 (60.8)	30/48 (62.5)
Physical exercise	39/79 (49.4)	5/39 (12.8)
Oral progestin	21/79 (26.6)	11/21 (52.4)
Levonorgestrel IUD	7/79 (8.9)	2/7 (28.6)
IM medroxyprogesterone Acetate	5/79 (6.3)	1/5 (20)
Morphin	4/79 (5.1)	1/4 (25)
Pelvic floor physiotherapy	4/79 (5.1)	2/4 (50)
Buscopan	2/79 (2.5)	2/2 (100)

NSAID = non-steroidal anti-inflammatory drugs, IM = intramuscular, IUD = intra-uterine device

## Discussion

In this exploratory study of trans adolescents assigned female at birth, almost four in five (77.5%) respondents reported pelvic pain in the last 6 months. The frequency of pelvic pain was lower in individuals using testosterone (69.4%) compared to those who were not (90%), but interpreting this finding must be done with caution given the exploratory nature of the study, the low number of participants, and the high risk of recruitment bias due to convenience sampling. Similar to other studies using convenience sampling (Grimstad et al., 2020; Zwickl et al., 2023), the rate of pelvic pain may have been over-estimated in this study and thus prevent us from making any conclusions about a true prevalence rate within the broader population.

Nonetheless, one of the strengths of our study was its inclusion of trans individuals who were not on testosterone therapy. Previously, only two other studies of pelvic pain in trans males have done so (Ferrando et al., 2021; Grimstad et al., 2023). However, as noted earlier, Grimstad et al. relied upon a retrospective chart review to assess the rate of pelvic pain in their subjects prior to starting testosterone. Meanwhile, only 10% (7 of 67) of the adult respondents of Ferrando et al were not on testosterone. In contrast, 40% of our study’s participants were not on testosterone which allowed us to observe that testosterone use was associated with a lower rate of pelvic pain among our participants. This lower rate was surprising given previous suggestions that testosterone was likely to increase the risk of pelvic pain (Grimstad et al., 2020; Zwickl et al., 2023). Although this was only an exploratory study and we cannot exclude a potential recruitment bias whereby individuals who had pelvic pain and were not using testosterone responded at a higher rate than those who had pain and were on testosterone, there are plausible reasons why testosterone use might be associated with reduced rates of pelvic pain. For example, testosterone treatment has been shown to exert anti-inflammatory effects, and androgens have been suggested as a potential strategy for chronic pelvic pain (Evans et al., 2021). At the same time, testosterone is known to inhibit ovulation and menstruation (Taub et al., 2020), and pelvic pain arising due to the menstrual cycle is likely to be attenuated in the context of testosterone treatment. Moreover, testosterone treatment has been associated with a significant improvement in trans men with other forms of chronic pain (Aloisi et al., 2007), leading to the suggestion that it may have direct neuromodulatory effects (Craft et al., 2004). Finally, gender-affirming testosterone therapy has been shown to reduce gender dysphoria (Nolan et al., 2023) and we hypothesize that—in the context of our study—adolescents receiving testosterone may be less likely to perceive and/or report pelvic pain given reduced levels of discomfort with the gendered aspects of their bodies. Consistent with the above observations, we found that 21.4% of our participants reported an improvement in their preexisting pelvic pain after commencing testosterone, while another study in transmasculine adults reported that almost one third no longer experienced pelvic pain after starting testosterone (Grimstad et al., 2023). Nevertheless, it should be noted that almost a quarter of those on testosterone in our cohort reported a worsening of their preexisting pelvic pain after testosterone initiation, while orgasm-associated pelvic pain was much more common in individuals using testosterone compared with those who were not (58.8% vs 23.5%). Together, these observations suggest that the effects of testosterone on pelvic pain are likely to be heterogeneous, affecting individuals in different ways. Finally, it is important to highlight that our study was not designed to determine a causal effect between testosterone use and a reduction (or increase) in pelvic pain, and ultimately prospective longitudinal studies are required to better understand the impact of testosterone on pelvic pain in trans individuals.

In this study, the reported rate of pelvic pain in adolescents using testosterone was 69.4%, which is similar to rates reported by previous exploratory studies with analogue study design in trans adults using testosterone (Grimstad et al., 2020; Zwickl et al., 2023). In contrast, retrospective cohort studies among trans individuals using testosterone have reported lower prevalence rates of pelvic pain, ranging between 23.4 and 36% (Grimstad et al., 2023; Moussaoui et al., 2024). On the one hand, it is likely that prevalence of pelvic pain was under-estimated in previous retrospective cohort studies because of under-report by patients themselves, and insufficient exploration and documentation by healthcare providers. On the other hand, our current study is associated with a risk of recruitment bias, whereby individuals with pelvic pain may have been more likely to enroll than individuals without, which may lead to an over-estimate of prevalence of pain.

Trans adolescents who were not using testosterone had a reported rate of pelvic pain of 90% in this study, which is higher than reported in cisgender adolescents assigned female at birth (Armour et al., 2020; Sasamoto et al., 2023; Tabaac et al., 2022). Although this difference might be explained by potential recruitment bias in our study, it is also likely to be related to differences in how pelvic pain is defined across studies. Most previous studies report rates of acyclical pelvic pain (that is excluding dysmenorrhea), which range between 14–55% in cisgender female adolescents and young adults (Armour et al., 2020; Sasamoto et al., 2023; Tabaac et al., 2022), and up to 65% in those with endometriosis (Sasamoto et al., 2023). In our study, pelvic pain was defined as any abdominal pain in the lower half of the abdomen, *without excluding cyclical causes*, which is consistent with the definition provided by the American College of Obstetricians and Gynecologists and the International Association for the Study of Pain (“Chronic Pelvic Pain,” 2020; *International Association for the Study of Pain. Classification of Chronic Pain, Second Edition (Revised).*, n.d.). This may have contributed to the high rate of pelvic pain observed in our study, since it is likely that respondents included the experience of dysmenorrhea in their report of pelvic pain. Consistent with this, previous studies have estimated that dysmenorrhea affects 71–92% of the cisgender female population (Armour et al., 2019, 2020; Schoep et al., 2019), which is in keeping with the rate of pelvic pain we observed in our adolescents not using testosterone. To better facilitate comparison between studies, future research in this area should ideally assess cyclical and acyclical pelvic pain separately.

Very few studies have explored pelvic pain in trans adolescents (Moussaoui et al., 2024; Shim et al., 2020) despite the impact of pelvic pain on quality of life, school participation, social activities and mental health in the general adolescent population having been well documented (Gallagher et al., 2018; Munro et al., 2023). In this study, half of participants reported missing school or work because of pelvic pain, and more than two thirds reported missing extracurricular activities because of pain. This contributes unfortunately to gender inequity in terms of academic performance, sport participation and psychosocial development.

Characteristics of pain, such as its main location to the suprapubic region, and its most common description as cramping, were consistent with previous literature (Grimstad et al., 2020; Moussaoui et al., 2024; Zwickl et al., 2023) and similar between adolescents using testosterone and those who were not. Sexual activity, including masturbation, orgasm, and vaginal or anal penetration, were commonly reported as a trigger of pain by our participants, which has also been reported in previous studies on trans adolescents and adults (Grimstad et al., 2020; Moussaoui et al., 2024; Zwickl et al., 2023). Participants using testosterone were more likely to report orgasm as a trigger for pelvic pain than individuals who were not using testosterone. Consistent with this, Zwickl et al. found higher odds of experiencing pelvic pain after testosterone initiation in individuals reporting pain with orgasm compared to those who were not (Zwickl et al., 2023). There is no clear explanation for this association between pain with orgasm and pelvic pain in trans individuals using testosterone, but pelvic floor muscles dysfunction or clitoral hypertrophy might contribute to it. In this study, medication use for menstrual suppression was associated with a higher chance of experiencing pelvic pain, which was also reported in a previous cohort study (Moussaoui et al., 2024). Nevertheless, after adjusting for testosterone status, the difference was no longer significant in our cohort. It should be highlighted that menstrual suppression medication is not only used to reduce gender dysphoria in this population, but also to relieve dysmenorrhea. Therefore, individuals with dysmenorrhea may be more likely to receive medication for menstrual suppression, which may be considered a confounding factor for pelvic pain.

Finally, our study showed a variety of treatment options and strategies used by trans adolescents to relieve pelvic pain, with variable reported rates of success. Paracetamol and NSAIDs were the most common treatments used and were reported as effective in approximately half of the users, which is roughly similar to previous studies (Moussaoui et al., 2024; Zwickl et al., 2023). Heat was commonly used and reported as effective in relieving pain in two thirds of the users, which has also been described by Zwickl et al. albeit to a lesser degree (Zwickl et al., 2023). A quarter of individuals with pelvic pain tried oral progestins and half of them found them helpful, which is higher than previously reported (Moussaoui et al., 2024). Pelvic floor physiotherapy was only attended by four individuals, with an effectiveness rate of 50%. It is unknown whether physiotherapy was not offered to other participants, or whether this small number reflects barriers to attend pelvic floor physiotherapy in trans masculine individuals. In our previous study on trans masculine adolescents, we found that only one out of five trans adolescents with pelvic pain to whom pelvic floor physiotherapy had been suggested attended the sessions (Moussaoui et al., 2024). Given the wide range of treatments used and the limited number of participants, this study does not allow us to draw any conclusions about the effectiveness of specific treatment options with confidence, especially those which were used by only a small number of adolescents. This lack of standardized approaches to manage pelvic pain has also been highlighted by others and reflects the still poorly understood mechanisms underlying pelvic pain in trans individuals (Grimstad et al., 2020; Zwickl et al., 2023).

Apart from the limitations already mentioned, there are other limitations of our study that are worth mentioning. Firstly, the response rate was low and the final number of participants is small, limiting the generalizability of our findings and potentially causing an over-estimation of the rate of pelvic pain in our study. One possible explanation for the low response rate is that email addresses to which study invitations were sent are not routinely updated, so some invitations may never have reached their intended recipients. Another plausible reason is that the vast majority of emails were sent to parents/guardians, raising the strong likelihood that these emails were never shared with their child. Moreover, trans young people are increasingly being invited to participate in research studies, and this may have reduced motivation to complete our survey. This low rate of participation and the high risk of recruitment bias (related to convenience sampling) prevent us from generalizing these findings and from providing a true estimate of the prevalence rate of pelvic pain in a broader population. Therefore, the interpretation of rates of pelvic pain among those using testosterone and those who were not must remain cautious. Secondly, we advertised the study among trans adolescents presenting to a specialized gender service in a tertiary pediatric hospital, therefore our findings cannot be generalized to the broader trans adolescent population. Thirdly, although the survey was designed by a group of healthcare professionals with expertise in transgender health, it is not a validated instrument and it is possible that respondents may have misinterpreted questions. Finally, all information was collected cross-sectionally and relied upon self-report, raising the possibility of recall bias.

## Conclusion

This study showed that pelvic pain is frequently reported by trans adolescents. The proportion of pelvic pain was lower in individuals who were using testosterone than in those who were not, but this finding must be interpreted cautiously given the exploratory nature of this study, the low number of participants and the risk of recruitment bias. While the description of pelvic pain was broadly similar between those using testosterone and those who were not, participants using testosterone were more likely to report orgasm as a trigger for pelvic pain than individuals who were not using testosterone. Pelvic pain had a significant impact on the lives of adolescents, with half of them reporting school or work absenteeism because of pain. The etiology of pelvic pain in trans individuals is still poorly understood and optimal management is uncertain. Prospective longitudinal studies are required to better understand the development and evolution of pelvic pain in trans adolescents.

## Supplementary Material

Supplemental Material
