# Supplementary material for: Is there an association between pelvic pain and gender-affirming testosterone therapy in trans masculine adolescents? An exploratory cross-sectional study
Source: Int J Transgend Health. 2024 Aug 20;27(2):636–46. doi: 10.1080/26895269.2024.2392685 (PMC12542325; doi:10.1080/26895269.2024.2392685)
Supplement: Supplemental Material [file WIJT_A_2392685_SM6003.docx]

**Survey on pelvic pain in trans adolescents**

1. **Patient information:**
2. “What is your gender identity?”
   - Male
   - Female
   - Non-binary
   - Different identity (specify): *free text answer*
3. “What is your sexual orientation?”

- Heterosexual
- Gay/Lesbian
- Bisexual
- Queer
- Different orientation (specify): *free word answer*
- Prefer not to disclose

1. **Hormone treatment:**
2. Have you ever been on testosterone?

- Yes
- I have been on testosterone in the past, but not currently
- No

1. For those who answered yes or I have been on testosterone in the past but not currently to question 2a: “How long have you been on testosterone?”

- Less than 3 months
- Between 3 and 6 months
- Between 6 and 12 months
- Between 1 and 2 years
- More than 2 years

1.
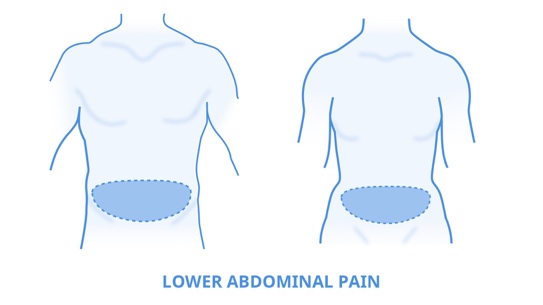
**Pelvic pain:**
2. “Over the last 6 months, have you experienced pelvic pain (which means abdominal pain located in the lower half of the abdomen, below the navel)?”
   - Yes
   - No (skip to section 4)
3. “How would you describe this pain?” (multiple answers allowed)
   - Cramping
   - Aching
   - Stabbing
   - Sharp
   - Throbbing
   - Hot/burning
   - Other (specify): *free word answer*
4.
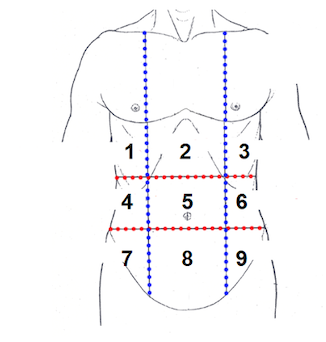
“Where is the pain located?” (multiple answers allowed)
   - - - - 1
         - 2
         - 3
         - 4
         - 5
         - 6
         - 7
         - 8
         - 9

*https://teachmeanatomy.info/abdomen/muscles/abdominal-wall/*

1. “On average, what is the severity of the pain?”

- scale 0-10 (0 = light to 10 = excruciating)

1. “Does the pain prevent you from going to school/work?”
   - Always
   - Often
   - Sometimes
   - Never
2. “Does the pain prevent you from participating in extracurricular activities (e.g. seeing friends, exercising, playing music)?”
   - Always
   - Often
   - Sometimes
   - Never

g. “What treatment or strategies (if any) have eased your pelvic pain?” (multiple answers allowed)

- - Paracetamol, panadol
  - Anti-inflammatory (NSAIDs, *ibuprofen, nurofen, naproxen, ponstan*, etc.)
  - Morphine-based medication (morphine, *tramadol*, *codeine*, etc.)
  - Heat
  - Exercise
  - Pelvic floor physiotherapy
  - Oral progestogen: *primolut*, *provera*
  - Intra-muscular injection progestogen: *depo-provera*
  - *Mirena* or intrauterine device
  - Injection of gonadotrophin releasing hormone agonists (e.g. *Zoladex, Lucrin, Triptorelin*)
  - Danazol
  - Laparoscopy/abdominal surgery
  - Other (specify): *free text answer*

1. “What treatments or strategies have you tried that have not worked?” (multiple answers allowed)
   - Paracetamol, panadol
   - Anti-inflammatory (NSAIDs, *ibuprofen, nurofen, naproxen, ponstan*, etc.)
   - Morphine-based medication (morphine, *tramadol*, *codeine*, etc.)
   - Heat
   - Exercise
   - Pelvic floor physiotherapy
   - Oral progestogen: *primolut*, *provera*
   - Intra-muscular injection progestogen: *depo-provera*
   - *Mirena* or intrauterine device
   - Injection of gonadotrophin releasing hormone agonists (*zoladex*)
   - Danazol
   - Laparoscopy/abdominal surgery
   - Other (specify): *free text answer*

The following three questions are on masturbation and sexual activity. You can skip them if you find them distressing. If they do not apply to you, you have the option of answering “not applicable”.

Relationship with sexual activity:

- 1. “Does touching of your external genitalia (including masturbation) cause pain?”
     - Always
     - Often
     - Sometimes
     - Never
     - Not applicable

1. “Do penetrative sexual activities (which means penetration of the vagina or anus by a penis, fingers or sex toys) provoke pain?”
   - - Always
     - Often
     - Sometimes
     - Never
     - Not applicable
2. “Does orgasm cause pain?”

- Always
- Often
- Sometimes
- Never
- Not applicable

1. For those who answered yes to question 2a: “In relation to testosterone initiation, do you think that”:

- This pelvic pain occurred for the first time after you started testosterone
- This pelvic pain was already present before you started testosterone, but has worsened since
- This pelvic pain was already present before you started testosterone, and has not changed since
- This pelvic pain was already present before you started testosterone, and has improved since
- This pelvic pain has disappeared since you have started testosterone

1. For those who answered “this pelvic pain occurred for the first time after I started testosterone” or “this pelvic pain was already present before I started testosterone but has worsened since” to question 3g: “When did you notice the onset or worsening of pain in relation to testosterone initiation?”
   - In the first month after testosterone initiation
   - Between 1 and 3 months after testosterone initiation
   - Between 3 and 6 months after testosterone initiation
   - Between 6 and 12 months after testosterone initiation
   - More than 1 year after testosterone initiation
2. **Periods :**
3. Have you ever had your periods?

- Yes
- No

1. Have you ever been on puberty blockers to stop your puberty from progressing (e.g. *Zoladex*, *Lucrin, Triptorelin*)?

- Yes
- No
- Unsure

1. If the answer is yes to question 4b: “Are you still on puberty blockers now?”

- Yes
- No

1. If the answer is yes to question 4a: “Have you ever been on medications to suppress your periods?”
   - Yes
   - No
2. “Have you ever been on one of these medications? Tick all the options that apply.” (multiple answers allowed)
   - - - *Primolut* or *Provera* (oral)
       - *Depo-provera* (intra-muscular injection)
       - *Mirena* or intrauterine device
       - *Implanon* or arm implant or the rod
       - Oral combined pill or birth control pill
3. If/for any medication ticked under question 4e: “Are you still on this medication now?”

- Yes
- No

1. For those who answered yes to question 2a: “Since starting testosterone, have your periods/bleeding stopped?”
   - Yes
   - No
   - Not applicable (I never had my periods)
2. If answer yes to question 4f: “How long did it take for your periods/bleeding to stop after starting testosterone?”
   - - - Less than 3 months after testosterone initiation
       - Between 3 and 6 months after testosterone initiation
       - Between 6 and 12 months after testosterone initiation
       - More than 1 year after testosterone initiation
3. If answer no to question 4f: “How much persistent bleeding do you experience?”
   - - - Spotting less than 1 day per month
       - Spotting more than 1 day per month
       - Heavy bleeding (like a period) less than 1 day per month
       - Heavy bleeding (like a period) more than 1 day per month
4. For those who answered yes to question 4a: “Have you ever experienced pain with your periods (period pain, pelvic pain, low back pain)?”
   - Always
   - Often
   - Sometimes
   - Never
5. For those who answered always, often or sometimes to question 4i: “On average, what was the severity of your period pain?”
   - - - scale 0-10 (0 = light to 10 = excruciating)
6. For those who answered yes to question 4a: “Have you ever experienced heavy periods?”
   - Always
   - Often
   - Sometimes
   - Never
7. **Clinical information:**
8. “What is your weight?”

- Free answer (kg)

1. “What is your height?”

- Free answer (cm or feet/inch)

1. “Have you ever been diagnosed with one of these conditions? Tick all that apply.” (multiple answers allowed)
   - Post-traumatic stress disorder (PTSD)
   - Autism spectrum disorder (ASD)
   - Depression
   - Anxiety
   - Endometriosis
   - Fibromyalgia
   - Chronic headache or migraine
   - Back pain
   - Other chronic pain condition (specify): free text
2. **Other**
   1. “Did your parent/caregiver supervise your response to this questionnaire?”

- Yes, my parent/caregiver supervised my answers to this questionnaire
- No, I completed this questionnaire on my own
